# Calycosin Improves Intestinal Mucosal Barrier Function after Gastrectomy in Rats through Alleviating Bacterial Translocation, Inflammation, and Oxidative Stress

**DOI:** 10.1155/2022/7412331

**Published:** 2022-06-26

**Authors:** Hui Peng, Lei Jin, Qi Zhang, Yi Shen, Zhen Wang, Fuhai Zhou, Qingsheng Yu

**Affiliations:** Department of General Surgery, The First Affiliated Hospital of Anhui University of Chinese Medicine, Hefei 230031, Anhui, China

## Abstract

**Objective:**

Calycosin is the main bioactive extract of *Astragali Radix* with anti-inflammation, antioxidant, and anticancer properties. Here, our study evaluated the protective effects and mechanisms of calycosin on intestinal mucosal barrier under gastrectomy.

**Methods:**

After receiving gastrectomy, the rats were administrated with 20 mg/kg, 40 mg/kg, or 80 mg/kg calycosin. Endotoxin, bacterial translocation, and intestinal bacterial flora were assayed. Intestinal injury was detected via hematoxylin and eosin staining. Tight junction indicators (occludin, claudin, and ZO-1) and apoptotic proteins (Bax, Bcl-2, and cleaved caspase 3) were examined in intestinal tissues. Inflammatory indicators (IL-1*β*, IL-6, and TNF-*α*) were examined in serum or intestinal specimens via ELISA. Apoptosis was assessed via TUNEL staining. IgA + B cells in intestinal tissues and sIgA in intestinal lumen were examined through immunohistochemistry and ELISA, respectively. Oxidative stress indicators (TSH, SOD, CAT, GSH-Px, and MDA) were also detected via ELISA.

**Results:**

Our results showed that calycosin administration decreased endotoxin levels in peripheral blood, intestine, and portal vein blood; lowered the bacterial translocation ratio; and regained the balance among intestinal bacterial flora (comprising bifidobacterium, lactic acid bacillus, enterobacter, enterococcus, aerobic bacteria, and anaerobic bacteria) in the rats with gastrectomy. After calycosin treatment, intestinal mucosal damage of the rats with gastrectomy was ameliorated, with the increase in expression of tight junction proteins. Additionally, calycosin reduced intestinal inflammation, apoptosis, secretion of sIgA, and oxidative stress in the rats with gastrectomy.

**Conclusion:**

Altogether, our findings demonstrate that calycosin may improve intestinal mucosal barrier function under gastrectomy via reducing bacterial translocation, inflammation, and oxidative stress.

## 1. Introduction

The gut is the largest reservoir of bacteria and endotoxin in the human body [1]. Normally, bacteria are in balanced proportions and functionally coordinated. To maintain the normal function of the intestinal mucosal biological barrier, in addition to maintaining the microecological balance, it is also necessary to maintain the normal intestinal mucosal mechanical and immune barriers [2, 3]. Under abnormal circumstances, especially when the body is subjected to severe stress conditions such as major surgery (especially gastrectomy), trauma, infection, and other pathophysiological processes such as ischemia-reperfusion, the bacteria in the intestinal lumen are displaced, triggering the intestinal mucosal barrier [4–6]. Gut-derived systemic infection, sepsis, systemic inflammatory response syndrome (SIRS), and multiple organ dysfunction syndrome (MODS), etc., seriously affect patients' clinical outcomes [7, 8]. Hence, effective approaches for preventing and treating intestinal barrier dysfunction are urgently needed.

Calycosin is an isoflavone compound and the main active component of traditional Chinese medicine *Astragali Radix* [9], which is a traditional Chinese medicine that has been broadly applied in treating cardiovascular diseases [10], idiopathic pulmonary fibrosis [11], cancers [12, 13], etc. For instance, experimental evidence suggests that calycosin can alleviate allergic contact dermatitis via repairing epithelial tight junctions, suppressing epithelial-derived initiative key factors, and maintaining epithelial barrier [14]. Nevertheless, the effects and mechanisms of calycosin on intestinal mucosal barrier under surgical trauma remain indistinct. To fill the gap, our study was conducted to demonstrate whether calycosin may improve intestinal mucosal barrier function after gastrectomy.

## 2. Materials and Methods

### 2.1. Experimental Design

Healthy male Wistar rats (300 ± 10 g, 3 months old) were provided by the Zhejiang Weitong Lihua Experimental Animal Technology Co., Ltd. (China). All rats were adaptively fed under environment conditions (temperature 22°C ± 1, humidity 55 ± 10%, along with a twelve-hour day/night cycle) for one week before the experiment began. Sixty rats were randomly separated into six groups: control group, sham operation group, model group, 20 mg/kg calycosin group, 40 mg/kg calycosin group, and 80 mg/kg calycosin group. For the control group, no treatment was given, and rats were free to drink and eat after the operation, without enteral nutrition and calycosin. For the sham operation group, only midline abdominal incision and suture were given, without gastrectomy; after the operation, the rats were given free access to water and food, without enteral nutrition and calycosin. For the model group, the rats received gastrectomy. Then, they were placed in metabolic cages, and the cages were free to move after they were awake, with fasting on the day of operation. From the first day, the silicone tube was connected to the microinjection pump and injected into the small intestine at a slow and uniform speed for 7 consecutive days. 160 g enteral nutrition Fresubin (Huarui Pharmaceutical Co., Ltd., China) was dissolved into 500 mL of solution with warm boiled water, and each 100 mL contained 830 kJ calorie. Each rat was provided with calorie at 120 kJ/(kg·d), and the formula for the amount of enteral nutrition injected every day was as follows: nutrient solution (ml/d) = (120 kJ/kg × body weight (kg))/8.3 kJ/ml. For 20, 40, or 80 mg/kg calycosin groups, the gastrectomy treatment, infusion method, and course of treatment were consistent with those in the model group. The rats were given 20, 40, or 80 mg/kg calycosin via the microinjection pump. All rats were euthanized with 200 mg/kg pentobarbital through intraperitoneal injection at the end of experiment.

The procedures of rat gastrectomy were as follows: after anesthetizing, the surgical field was routinely sterilized, and a midabdominal incision was made to enter the abdomen. Partial resection of the anterior wall between the greater and greater curvatures of the gastric body was performed, and 1-0 silk sutures were used to suture the full-thickness layer and the seromuscular layer. Meanwhile, a 12-gauge syringe needle was used to poke a hole in the anterior wall of the jejunum at a distance of 15 cm from the ligament of Treitz. A silicone catheter with a diameter of 1.0 mm was placed in the jejunum and fixed by double-layer purse-string suture. The end of the catheter was pulled out through the abdominal wall. Through the subcutaneous tunnel, it was led out from the skin of the bilateral scapular region on the back of the neck and fixed with a spring protection device. All animal experimental procedures were approved by the Animal Ethics Committee of the First Affiliated Hospital of Anhui University of Chinese Medicine (KY-2021-008).

### 2.2. Endotoxin Assay

After strict aseptic operation, the portal vein was found at the root of the mesentery, and the proximal end of the oral vein was ligated with 4-0 silk thread. After the portal vein was filled, about 0.3 ml of blood was collected from the portal vein with a 1 ml sterile syringe, placed in an anticoagulant EP tube, and stored at 4°C. Meanwhile, rat peripheral blood was collected. Serum was separated and treated with thermodilution. The contents of the ileum and cecum were separately collected, weighed, mixed with pyrogen-free saline, and centrifuged at 5000 g for 10 min at 4°C. The supernatant was serially diluted 10-fold with pyrogen-free water. Endotoxin levels in portal vein blood peripheral blood, and intestinal samples were assayed utilizing matrix limulus kit following the manufacturer's specification (130312; Xiamen Limulus Reagent Experiment Factory Co., Ltd., China).

### 2.3. Bacterial Translocation Assay

After the rats were anesthetized, the abdominal cavity was opened under sterile conditions, and the mesenteric lymph nodes, liver, kidney, lung, intestine, and spleen were collected. 0.5 g of each organ was suspended in 1 mL of standard saline solution and homogenized. Then, 0.5 mL of the homogenate was inoculated in 10 cm cell culture plates at 37°C lasting 48 h for measuring bacterial growth. If the number of bacterial colonies was greater than >100 CFU/g, it was counted as positive for bacterial culture. The bacterial translocation rate was calculated as the number of positive organs for bacterial culture/total number of cultured organs.

### 2.4. Detection of Intestinal Bacterial Flora

Using the JLQ-S1 colony counter, the bacteria were identified according to the morphological characteristics of bacteria. Rat cecal tissues (0.5–1.0 cm) were dissected longitudinally, and the contents were gently removed and placed in a large test tube with irrigation fluid. The samples were washed 3 times with 0.01 mol/L PBS, put into a new test tube containing 2 ml of normal saline, and homogenized. 100 *μ*l of each homogenate was inoculated on TPY agar medium, cultured in an anaerobic environment for 72 h, and the bifidobacteria were identified and enumerated by the anaerobic chamber method. LC medium and under aerobic conditions were used for the identification and enumeration of lactic acid bacteria. EMB medium was used to identify and count the number of enterobacterium. EC medium was used to identify and count the number of enterococci. BA and BHI blood agar media were used to analyze the total number of aerobic and anaerobic bacteria, respectively. The number of bacteria per gram of feces (CFU/g) = the number of colonies×10^*n*^ (10^*n*^: the dilution corresponding to the number of colonies).

### 2.5. Hematoxylin and Eosin Staining

About 3 cm of small bowel tissue above 2 cm from the ileocecal area was fixed with 10% formalin, paraffin embedded, and sectioned. After dewaxing with xylene, a series of ethanol was used for hydration. The sections were stained with hematoxylin (B600020; Proteintech, China) for 5 min. After washing with alkaline PBS and bluing for 2 min, they were rinsed with running water for 3 min. Afterwards, they were stained with eosin (Sigma, USA) for 10 min and flash washed with distilled water. Neutral gums were used for coverslipping. The morphological and structural changes of intestinal mucosal epithelium were observed under an optical microscope (eclipse Ci-e; Nikon, Japan). According to the results of hematoxylin and eosin staining, the degree of intestinal injury was evaluated by Chiu's score [15].

### 2.6. Real-Time qPCR

100 mg of small intestinal tissues were pulverized in liquid nitrogen and collected into Eppendorf tubes. Trizol reagent (10606ES60; Yeasen, Shanghai, China) was used to extract total RNA. The optical density value was measured in a UV spectrophotometer, and the RNA concentration and content were calculated. The reverse transcription cDNA kit was used for mRNA reverse transcription reactions. Real-time qPCR was performed by real-time fluorescence quantitative method. The data were processed by 2^−ΔΔCT^ method, and the mRNA expressions of occludin, claudin, and ZO-1 were examined. The primer sequences are listed in Table 1.

### 2.7. Western Blot

100 mg of small intestinal tissues were pulverized in liquid nitrogen and collected into Eppendorf tubes. The extracted total protein was separated by polypropylene gel electrophoresis and transferred to nitrocellulose membrane and labeled with primary antibody of occludin (1 : 3000; 27260-1-AP; Proteintech, China), claudin (1 : 3000; 13050-1-AP; Proteintech, China), ZO-1 (1 : 1000; 21773-1-AP; Proteintech, China), Bax (1 : 1000; 60267-1-Ig; Proteintech, China), Bcl-2 (1 : 1000; ABP50759; Abbkine, USA), cleaved caspase 3 (1 : 1000; 66470-2-Ig; Proteintech, China), GAPDH (1 : 5000; 60004-1-Ig; Proteintech, China), and secondary antibody (1 : 10000; SA00001-2; Proteintech, China). Images were visualized with chemiluminescence method and acquired by an automated gel imaging system (Bio-rad, USA). The protein content of occludin, claudin, and ZO-1 was quantitatively analyzed by gel image analysis software.

### 2.8. Inflammatory Cytokine Assay

Enzyme-linked immunosorbent assay (ELISA) kits for interleukin (IL)-1*β* (KET9001), IL-6 (KET9004), and tumor necrosis factor-*α* (TNF-*α*; KET9007) were purchased from Abbkine company (Wuhan, China). The levels of IL-1*β*, IL-6, and TNF-*α* were assayed in serum and intestinal specimens following the manufacturer's instructions.

### 2.9. Apoptosis Assay

Apoptosis of intestinal epithelial cells was assayed with terminal deoxynucleotidyl transferase dUTP nick end labeling (TUNEL) staining kit (ATK00001, Atagenix, Wuhan, China) following the manufacturer's instructions.

### 2.10. Immunohistochemistry

Intestine tissues were fixed in 4% paraformaldehyde and embedded in paraffin following dehydration. Afterwards, tissues were sectioned into 5 *µ*m, dewaxed in xylene, and rehydrated through ethanol reagent (C1032; Solarbio, China). 3% H_2_O_2_ (10011218; Sinopharm Chemical Reagent Co., Ltd, China) was utilized for blocking endogenous peroxidase. Then, heat-induced antigen retrieval was carried out with citrate buffer. After being blocked with goat serum, the sections were incubated overnight at 4°C with immunoglobulin A (IgA) antibodies (1 : 5000; 11449-1-AP; Proteintech, Wuhan, China). Images were acquired under the optical microscope (eclipse Ci-e; Nikon, Japan).

### 2.11. Secretory IgA (sIgA) Assay

Small intestine tissues from the ligament of Trevor to the ileocecal region were collected, and the blood stains and feces were washed with normal saline. The intestinal cavity was washed with 10% acetic acid solution. The lavage fluid was collected and centrifuged at 20000 r/min for 30 min at 4°C. The supernatant was collected and stored at −70°C for sIgA detection using ELISA kit (ED-30356; Xiamen Lunchangshuo Biotechnology Co., Ltd., China).

### 2.12. Antioxidant and Oxidant Assay

Intestinal tissues were homogenized, centrifuged, and the supernatant was harvested. Antioxidant indicators (total sulfhydryl (TSH), superoxide dismutase (SOD), catalase (CAT), and glutathione peroxidase (GSH-Px)) and oxidant indicator (malonaldehyde (MDA)) were conducted utilizing reagent kits following the manufacturer's instructions (Nanjing Jiancheng, China).

### 2.13. Statistical Analysis

Data are calculated as the mean ± standard error, and GraphPad Prism software (version 8.0.1; GraphPad Software Inc., San Diego, CA, USA) was applied to perform statistical analysis. One-way analysis of variance was conducted for calculating the significant differences between the groups. *P* values <0.05 were considered significant.

## 3. Results

### 3.1. Calycosin Alleviates Gut Bacterial Translocation after Gastrectomy in Rats

**Figure 1:**
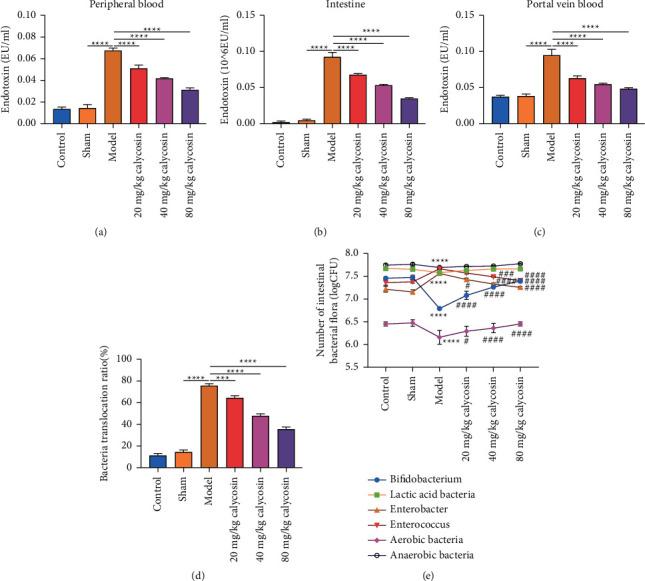
Calycosin alleviates gut bacterial translocation after gastrectomy in rats. (a–c) Endotoxin levels in peripheral blood, intestine, and portal vein blood samples from control, sham, model, and 20, 40, or 80 mg/kg calycosin group (^*∗∗∗∗*^*p* < 0.0001). (d) Bacteria translocation ratio of control, sham, model, and 20, 40, or 80 mg/kg calycosin group (^*∗∗∗*^*p* < 0.001; ^*∗∗∗*^^*∗*^*p* < 0.001). (e) Number of intestinal bacterial flora of control, sham, model, and 20, 40 or 80 mg/kg calycosin group (compared with sham group, ^*∗∗∗*^^*∗*^*p* < 0.001; compared with model group, #*p* <  0.05; ###*p* < 0.001; and ####*p* < 0.0001).

Endotoxin may promote intestinal mucosal barrier damage and bacterial translocation from the gut to systemic organs [16]. Hence, this study assessed the effects of calycosin on endotoxin of rats after gastrectomy. Our study showed that endotoxin levels were significantly increased in peripheral blood (Figure 1(a)), intestine (Figure 1(b)), and portal vein blood (Figure 1(c)) of the model rats with gastrectomy than of the sham-operated rats. 20, 40, and 80 mg/kg calycosin administration decreased endotoxin levels in peripheral blood (Figure 1(a)), intestine (Figure 1(b)), and portal vein blood (Figure 1(c)) of the model rats with gastrectomy, indicating that calycosin might improve intestinal mucosal barrier injury as well as bacterial translocation from the gut to systemic organs through lowering endotoxin levels of the rats with gastrectomy. We further investigated the effect of calycosin on bacterial translocation of the rats with gastrectomy. As shown in Figure 1(d), the bacterial translocation ratio was higher in the rats with gastrectomy compared with the sham-operated rats. 20, 40, and 80 mg/kg calycosin lowered the bacterial translocation ratio of the rats with gastrectomy. This indicated that calycosin enabled to alleviate the bacterial translocation among organs during gastrectomy. In Figure 1(e), we found that calycosin administration significantly regained the balance among intestinal bacterial flora (comprising bifidobacterium, lactic acid bacillus, enterobacter, enterococcus, aerobic bacteria, and anaerobic bacteria) in the rats with gastrectomy.

### 3.2. Calycosin Alleviates Intestinal Mucosal Damage of Rats with Gastrectomy

**Figure 2:**
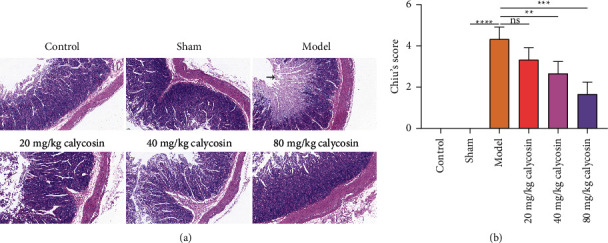
Calycosin alleviates intestinal mucosal damage of rats with gastrectomy. (a) Hematoxylin and eosin staining for the morphological and structural changes of intestinal mucosal epithelium from control, sham, model, 20, 40 or 80 mg/kg calycosin group (bar, 100 *μ*m). Magnification, 100x. The pathological injury sites are indicated with arrows. (b) Chiu's score for the degree of intestinal injury according to hematoxylin and eosin staining (ns: not significant; ^∗∗^*p* < 0.01; ^*∗∗∗*^*p* < 0.001; ^*∗∗∗∗*^*p* < 0.0001).

Hematoxylin and eosin staining results suggested that the intestinal tissue structure of the control or sham-operated rats was intact, and the intestinal villi were intact and slender (Figure 2(a)). The epithelial cells on the top of the intestinal villi were shed and necrotic in the rats with gastrectomy. After administration with calycosin, the height of the rat intestinal villi was significantly increased. Especially, we observed that rat intestinal villi were relatively intact under 80 mg/kg calycosin treatment. According to H&E staining, Chiu's score was used to evaluate the severity of intestinal mucosal injury. The rats with gastrectomy exhibited the dramatically increased Chiu's score than the sham-operated rats (Figure 2(b)). 40 and 80 mg/kg calycosin lowered Chiu's score of the rats with gastrectomy. Hence, calycosin was capable of maintaining intestinal mucosal barrier integrity of rats with gastrectomy.

### 3.3. Calycosin Maintains Intestinal Mucosal Barrier Integrity of Rats with Gastrectomy through Activating Occludin/Claudin/ZO-1 Signaling Pathway

**Figure 3:**
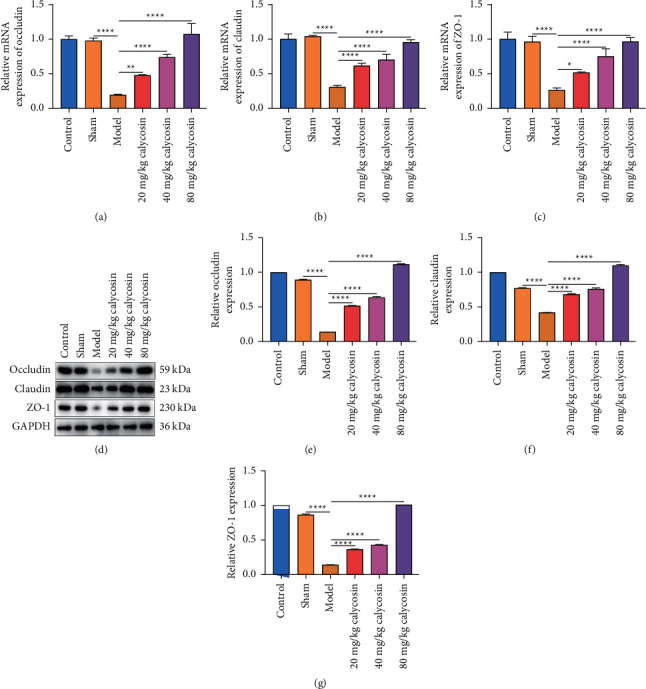
Calycosin maintains intestinal mucosal barrier integrity of rats with gastrectomy through activating the occludin/claudin/ZO-1 signaling pathway. (a–c) The mRNA level of occludin, claudin, or ZO-1 in the intestinal tissues of control, sham, model, and 20, 40, or 80 mg/kg calycosin group (^*∗*^*p* < 0.05; ^*∗∗*^*p* < 0.01; ^*∗∗∗∗*^*p* < 0.0001). (d–g) The protein level of occludin, claudin, or ZO-1 in intestinal tissues of control, sham, model, and 20, 40, or 80 mg/kg calycosin group (^*∗∗∗∗*^*p* < 0.0001).

Tight junction proteins—occludin, claudin, and ZO-1—are indispensable for maintaining the normal function of the gut barrier [17]. Their expression was examined in intestinal tissues. Our results demonstrated that occludin, claudin, and ZO-1 expression was significantly downregulated in the rats with gastrectomy in comparison with the sham-operated rats at the mRNA and protein levels (Figures 3(a)–3(g)). 20, 40, and 80 mg/kg calycosin dramatically elevated occludin, claudin, and ZO-1 expression in the rats with gastrectomy. Above findings indicated that calycosin might maintain intestinal mucosal barrier integrity of rats with gastrectomy through activating occludin/claudin/ZO-1 signaling pathway.

### 3.4. Calycosin Exerts Systemic and Intestinal Anti-Inflammatory Effects after Gastrectomy in Rats

**Figure 4:**
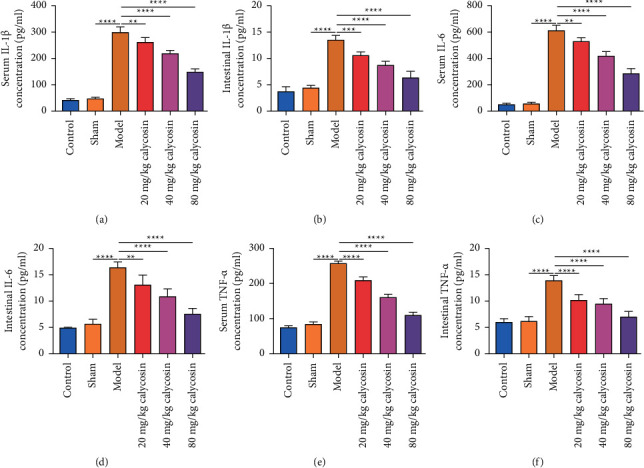
Calycosin exerts systemic and intestinal anti-inflammatory effects after gastrectomy in rats. (a–f) The level of inflammatory indicators IL-1*β*, IL-6, or TNF-*α* in serum or intestine specimens of control, sham, model, and 20, 40, or 80 mg/kg calycosin group (^*∗∗*^*p* < 0.01; ^*∗∗∗*^*p* < 0.001; ^*∗∗∗∗*^*p* < 0.0001).

To investigate the effects of calycosin on inflammatory response in rats with gastrectomy, we examined the levels of inflammatory indicators in serum and intestine specimens. Our results demonstrated that the levels of IL-1*β*, IL-6, and TNF-*α* were significantly increased in serum and intestine specimens of the rats with gastrectomy than that of the sham-operated rats (Figures 4(a)–4(f)). 20, 40, and 80 mg/kg calycosin dramatically lowered IL-1*β*, IL-6, and TNF-*α* levels in serum or intestine specimens of the rats with gastrectomy. Above data indicated that calycosin exerted anti-inflammatory effects after gastrectomy in rats.

### 3.5. Calycosin Decreases Apoptosis of Intestinal Epithelial Cells of Rats with Gastrectomy

**Figure 5:**
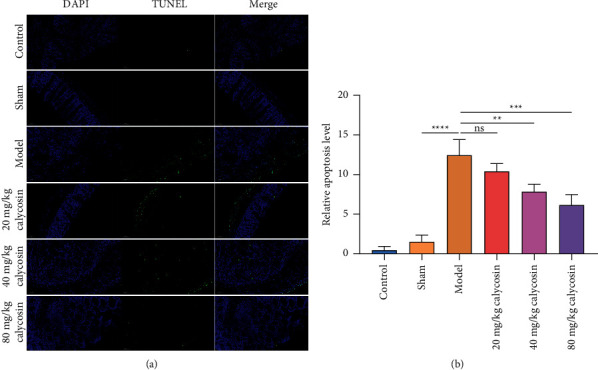
Calycosin decreases apoptosis of intestinal epithelial cells of rats with gastrectomy. (a) TUNEL staining of the apoptosis of intestinal epithelial cells of control, sham, model, and 20, 40, or 80 mg/kg calycosin group (bar, 50 *μ*m). Magnification, 200x. (b) Quantification of apoptotic levels of intestinal epithelial cells of control, sham, model, and 20, 40 or 80 mg/kg calycosin group (ns: not significant; ^*∗∗*^*p* < 0.01; ^*∗∗∗*^*p* < 0.001; ^*∗∗∗∗*^*p* < 0.0001).

**Figure 6:**
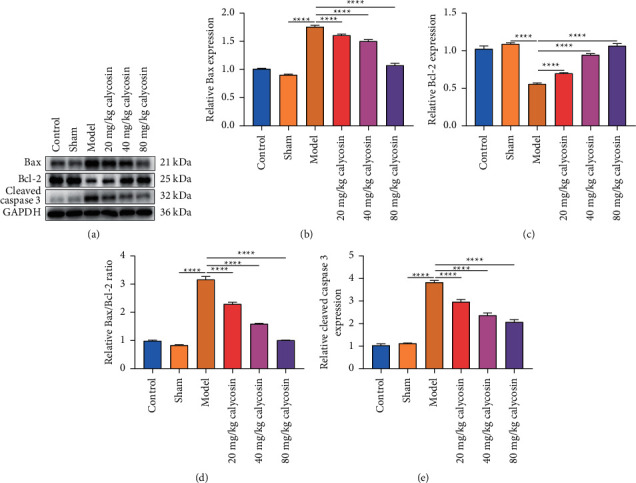
Calycosin reduces Bax and cleaved caspase 3 and increases Bcl-2 in intestine of rats with gastrectomy. (a) Western blot of the expression of Bax, Bcl-2, and cleaved caspase 3 in intestine tissues of control, sham, model, and 20, 40, or 80 mg/kg calycosin group. (b–e) The levels of Bax, Bcl-2, and Bax/Bcl-2 ratio along with cleaved caspase 3 in intestine tissues of control, sham, model, and 20, 40, or 80 mg/kg calycosin group (^*∗∗∗∗*^*p* < 0.0001).

TUNEL staining was conducted for evaluating whether calycosin alleviated the apoptotic levels of intestinal epithelial cells of rats receiving gastrectomy. In comparison with the sham-operated rats, the apoptotic levels of intestinal epithelial cells were significantly elevated (Figures 5(a) and 5(b)). 40 mg/kg and 80 mg/kg calycosin dramatically weakened intestinal epithelial cell apoptosis in rats with gastrectomy. Further analysis showed that Bax, Bax, and Bcl-2 along with cleaved caspase 3 levels were higher, and Bcl-2 levels were lower in the intestine of rats receiving gastrectomy than that of the sham-operated rats (Figures 6(a)–6(e)). But 20, 40, and 80 mg/kg calycosin administration reduced Bax, Bax/Bcl-2 ratio, and cleaved caspase 3 levels and significantly elevated Bcl-2 levels in intestinal tissues of the rats with gastrectomy. Altogether, calycosin may decrease apoptosis of intestinal epithelial cells of rats with gastrectomy.

### 3.6. Calycosin Promotes Secretion of sIgA on Intestinal Mucosal Surface of Rats with Gastrectomy

**Figure 7:**
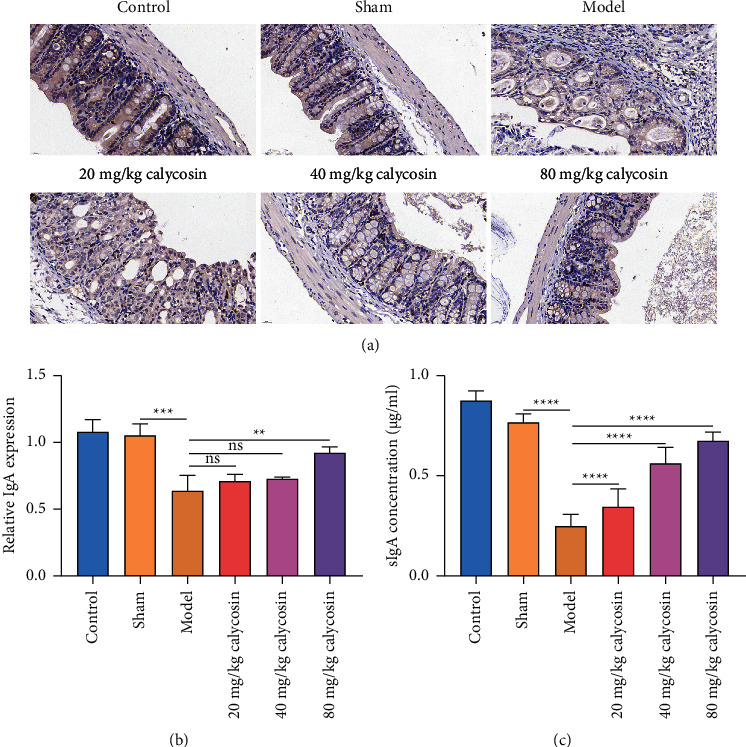
Calycosin promotes secretion of sIgA on intestinal mucosal surface of rats with gastrectomy. (a) Immunohistochemistry of IgA + B cells in intestine tissues of control, sham, model, and 20, 40 or 80 mg/kg calycosin group (bar, 20 *μ*m). Magnification, 200x. (b) Quantification of IgA expression in intestine tissues of control, sham, model, and 20, 40, or 80 mg/kg calycosin group (ns: not significant; ^*∗∗*^*p* < 0.01; ^*∗∗∗*^*p* < 0.001). (c) The concentration of sIgA in the intestinal lumen of control, sham, model, and 20, 40, or 80 mg/kg calycosin group (^*∗∗∗∗*^*p* < 0.0001).

Intestinal mucosal immune function was further evaluated. IgA + B cells were examined in intestine of rats with gastrectomy. In comparison with the sham-operated rats, IgA + B cells were significantly decreased in intestine tissues of the rats with gastrectomy (Figures 7(a) and 7(b)). 80 mg/kg calycosin significantly elevated the levels of IgA + B cells in intestine tissues of the rats with gastrectomy, indicating that calycosin improved intestinal mucosal immune barrier after gastrectomy. We also investigated that sIgA concentration was significantly lower in the intestinal lumen of the rats with gastrectomy than that of the sham-operated rats (Figure 7(c)). 20, 40, and 80 mg/kg calycosin elevated sIgA levels in the intestinal lumen of the rats with gastrectomy. Hence, calycosin promoted secretion of sIgA on intestinal mucosal surface of rats with gastrectomy.

### 3.7. Calycosin Alleviates Oxidative Stress in Intestine of Rats with Gastrectomy

**Figure 8:**
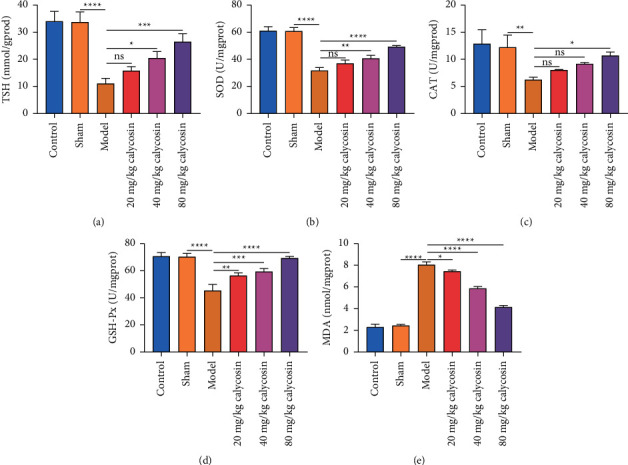
Calycosin alleviates oxidative stress in intestine of rats with gastrectomy. (a–e) The level of TSH, SOD, CAT, GSH-Px, or MDA in intestinal homogenates of control, sham, model, and 20, 40 or 80 mg/kg calycosin group (ns: not significant; ^*∗*^*p* < 0.05; ^*∗∗*^*p* < 0.01; ^*∗∗∗*^*p* < 0.001; ^*∗∗∗∗*^*p* < 0.0001).

Antioxidative stress indicators (TSH, SOD, CAT, and GSH-Px) and oxidative stress indicator (MDA) were examined in intestinal homogenates. In comparison with the sham-operated rats, TSH, SOD, CAT, and GSH-Px levels were lower, and MDA levels were higher in intestinal homogenates of the rats with gastrectomy (Figures 8(a)–8(e)), indicating oxidative stress damage occurred in the intestinal mucosa after gastrectomy. 40 and 80 mg/kg calycosin elevated TSH and SOD levels in intestinal homogenates of the rats with gastrectomy. Meanwhile, 80 mg/kg calycosin significantly increased the levels of CAT in intestinal homogenates of the rats with gastrectomy. Additionally, 20, 40, and 80 mg/kg calycosin elevated GSH-Px levels and reduced MDA levels in intestinal homogenates following gastrectomy. Altogether, calycosin alleviated oxidative stress in the intestine of rats with gastrectomy.

## 4. Discussion

The gut has been considered as the driver of crucial illness and organ damage [18]. Surgical trauma especially gastrectomy can result in intestinal mucosal barrier dysfunction, translocation of bacteria, and endotoxins in the intestinal lumen, leading to intestinal systemic infection and sepsis, and even SIRS and MODS, which seriously affects patients' clinical outcomes [19, 20]. Our study revealed the protective properties and mechanisms of calycosin on the intestinal mucosal barrier under surgical trauma.

The normal intestinal mucosal barrier comprises mechanical barrier, immune barrier, and chemical barrier along with biological barrier [21], which prevents harmful substances in the intestine, such as bacteria and toxins from passing through the intestinal mucosa and entering the structure and function of other tissues, organs, and blood circulation [22]. Mechanical and immune barriers play central roles in effectively blocking intestinal parasites and their toxins from passing through the intestinal mucosa as well as translocating to extraintestinal tissues and organs [23]. The intestinal mucosal biological barrier is an interdependent and interacting microecosystem composed of resident intestinal flora [24]. This huge and complex microecosystem has the functions of preventing the invasion of pathogenic bacteria, synthesizing vitamins and participating in the metabolism of certain substances [25]. Most of the intestinal bacteria are anaerobic bacteria such as lactobacillus and bifidobacteria, and there are less aerobic bacteria and facultative anaerobic bacteria [25]. Under normal circumstances, these bacterial groups maintain a stable ratio, forming an interdependent and interacting microecosystem with the host's microspatial structure [26]. Our results demonstrated that calycosin decreased endotoxin levels in peripheral blood, intestine, and portal vein blood; lowered the bacterial translocation ratio; and regained the balance among intestinal bacterial flora (comprising bifidobacterium, lactic acid bacillus, enterobacter, enterococcus, aerobic bacteria, and anaerobic bacteria) in the rats with gastrectomy, thereby maintaining the intestinal mucosal biological barrier. Consistently, previous evidence has demonstrated that *Astragali Radix* can improve gut microbiota balance and intestinal mucosal barrier [27].

The mechanical barrier comprises intestinal mucosal epithelial cells, intercellular tight junction, and mucus layer, as the normal mechanical barrier mucosal epithelial cells and various connecting structures between cells, such as tight junction, gap junction, and adhesion junction [28]. Among them, tight junctions play a key role in the mechanical barrier. The tight junction proteins are important for maintaining the structure and functions of tight junctions and the intestinal mucosal mechanical barrier [29]. Our results showed that calycosin significantly alleviated intestinal mucosal damage and increased the expression of occludin, claudin, and ZO-1 in intestinal tissues of the rats with gastrectomy and thus maintained intestinal mucosal mechanical barrier following gastrectomy.

The intestinal mucosal immune barrier is mainly composed of cell populations of gut-associated lymphoid tissue [30]. Structurally, it is mainly composed of collecting lymphoid nodules, intraepithelial lymphocytes, and lamina propria lymphocytes [31]. The former is the induction site of mucosal immunity, and the latter two are the effector sites of mucosal immunity [32]. Here, calycosin lowered the levels of inflammatory indicators (IL-1*β*, IL-6, and TNF-*α*) in serum as well as intestine specimens from the rats with gastrectomy, demonstrating the systemic and intestinal anti-inflammatory effects of calycosin. Moreover, calycosin increased the levels of IgA + B cells in intestinal tissues as well as the secretion of sIgA in intestinal lumen in the rats with gastrectomy. Hence, calycosin enabled to maintain intestinal mucosal immune barrier after gastrectomy.

Enterocyte apoptosis is an essential mechanism of intestinal barrier damage [33]. After calycosin administration, apoptosis of intestinal epithelial cells of the rats with gastrectomy was significantly alleviated. Additionally, calycosin lowered Bax, Bax/Bcl-2 ratio, and cleaved caspase 3 levels but elevated Bcl-2 levels in the intestine of the rats with gastrectomy. Oxidative stress represents the imbalance between pro- and antioxidants, leading to molecular or cellular injury [34]. Calycosin elevated the levels of antioxidative stress indicators (TSH, SOD, CAT, and GSH-Px) as well as decreased the levels of oxidative stress indicator (MDA) in intestinal homogenates of the rats with gastrectomy, demonstrating the antioxidative stress property of calycosin.

## 5. Conclusion

Altogether, our study suggested that calycosin improved intestinal mucosal barrier function after gastrectomy by lowering bacterial translocation, inflammation, and oxidative stress, revealing the protective properties and mechanisms of calycosin on the intestinal mucosal barrier under surgical trauma. Hence, calycosin displayed a promising practical application prospect for solving intestinal barrier damage in the future.

## Figures and Tables

**Table 1:** The primer sequence information for real-time qPCR.

Gene name	Primer sequence
GAPDH	5′-ACCTGTCGTGTAGTCGGTTT-3′ (forward)
	5′-CCCTGTTGCTGTAGCCATATT-3′ (reverse)

Occludin	5′-TCCAACGGCAAAGTGAATGG-3′ (forward)
	5′-ACCTGTCGTGTAGTCGGTTT-3′ (reverse)

Claudin 1	5′-GGACAACATCGTGACTGCTC-3′ (forward)
	5′-AGCCATCCACATCTTTTGCA-3′ (reverse)

ZO-1	5′-TATCCAAACCAGACCCACCC-3′ (forward)
	5′-GGCTTTGGTGTGAATCGGTT-3′ (reverse)

## Data Availability

The datasets analyzed during the current study are available from the corresponding author on reasonable request.

## Abbreviations

SIRS: Systemic inflammatory response syndrome
MODS: Multiple organ dysfunction syndrome
ELISA: Enzyme-linked immunosorbent assay
il: Interleukin
TNF-*α*: Tumor necrosis factor-*α*
TUNEL: Terminal deoxynucleotidyl transferase dUTP nick end labeling
SIgA: Secretory immunoglobulin A
TSH: Total sulfhydryl
SOD: Superoxide dismutase
CAT: Catalase
GSH-px: Glutathione peroxidase
MDA: Malonaldehyde.
